# Identification of coronary heart disease in asymptomatic individuals with diabetes mellitus to screen or not to screen

**Published:** 2015-03-30

**Authors:** Paco E Bravo, Bruce M Psaty, Marcelo F Di Carli, Kelley R Branch

**Affiliations:** 1 Division of Cardiology, Department of Medicine, University of Washington. Seattle, WA, USA; 2 Cardiovascular Health Research Unit, Departments of Medicine, Epidemiology, and Health Services, University of Washington. Seattle, WA, USA; 3 Noninvasive Cardiovascular Imaging Program. Departments of Radiology and Medicine,. Brigham and Women's Hospital, Harvard Medical School, Boston, MA, USA

**Keywords:** Screening, coronary heart disease, diabetes

## Abstract

Coronary heart disease (CHD) is highly prevalent in patients with diabetes mellitus (DM), and remains the single most common cause of death among this population. Regrettably, a significant percentage of diabetics fail to perceive the classic symptoms associated with myocardial ischemia. Among asymptomatic diabetics, the prevalence of abnormal cardiac testing appears to be high, ranging between 10% and 62%, and mortality is significantly higher in those with abnormal scans. Hence, the potential use of screening for CHD detection among asymptomatic DM individuals is appealing and has been recommended in certain circumstances. However, it was not until recently, that this question was addressed in clinical trials. Two studies randomized a total of 2,023 asymptomatic diabetics to screening or not using cardiac imaging with a mean follow up of 4.4 ± 1.4 years. In combination, both trials showed lower than expected annual event rates, and failed to reduce major cardiovascular events in the screened group compared to the standard of care alone. The results of these trials do not currently support the use of screening tools for CHD detection in asymptomatic DM individuals. However, these studies have important limitations, and potential explanations for their negative results that are discussed in this manuscript.

## Abstract (es)

La enfermedad de la arterias coronarias (EAC) es muy prevalente en pacientes con diabetes mellitus (DM), y continúa siendo la principal causa de muerte en estos pacientes. Desafortunadamente, muchos diabéticos pueden carecer de síntomas de alerta en la presencia de isquemia miocárdica, por lo cual el diagnóstico de EAC puede ocurrir de manera tardía. Estudios observacionales han sugerido que la prevalencia de isquemia miocárdica puede ser alta en diabéticos asintomáticos (10 al 62% según la serie) y la mortalidad es mayor en esos pacientes. Por esto, el uso de pruebas para detección de EAC en el paciente diabético asintomático parece atractivo y es recomendado en ciertas circunstancias. Sin embargo, no fue si no hasta hace poco que dos estudios investigaron el verdadero rol de estas pruebas de manera aleatoria. En conjunto, 2,023 pacientes diabéticos asintomáticos fueron aleatorizados a recibir o no una prueba para detección de EAC y fueron seguidos en promedio por 4.4 ± 1.4 años. Al final de seguimiento, ambos estudios mostraron menos eventos cardiovasculares de los esperados, y el uso de pruebas para detección de EAC no redujo la tasa de eventos cardiovasculares comparado al no uso de estas pruebas. Los resultados de estos ensayos clínicos no soportan actualmente el uso de estas pruebas en el paciente diabético asintomático. Sin embargo, estos estudios tienen limitaciones importantes, y posibles hipótesis para explicar los resultados que son discutidas en el artículo.

## Introduction

Diabetes mellitus (DM) is a major public health problem in the United States (U.S.) and worldwide. The age-adjusted U.S. prevalence (per 100 persons) of diagnosed DM cases has increased in the last two decades from 3.5 (95%CI, 3.2-3.9) in 1990 to 8.3 (95%CI, 7.9-8.7) in 2012 1. More concerning, the percentage of individuals with DM in 2012 was 16.2% for the age group 45-64 and 25.9% for people age 65 or older 2.

Diabetes is a major risk factor for the development of coronary heart disease (CHD) and its most severe complications myocardial infarction (MI), sudden cardiac death (SCD) and congestive heart failure (CHF) 3,4. Consequently, cardiovascular morbidity and mortality are disproportionately high among individuals with DM compared to those without. For instance, the age-adjusted rates of hospitalization for MI and cardiovascular deaths are 1.8 and 1.7 times higher respectively among adult diabetics than non-diabetic individuals 2. Moreover, a study published by Haffner *et al*., revealed that individuals with long-standing type 2 DM (mean duration 8.06 ±0.14 yrs), but without prior MI history, have similar rates of MI and cardiac deaths compared to non-diabetics with a previous MI 3. Appropriately, the U.S. guidelines for the management of lipids treat DM as a CHD risk-equivalent 5, and many care providers may have a low threshold for performing non-invasive as well as invasive testing to identify CHD among individuals with DM.

## Prevalence of sub-clinical coronary heart disease among diabetics

Individuals with DM appear to be less able to perceive some of the classic symptoms associated with ischemia, and may have asymptomatic "silent myocardial ischemia". This can eventually manifest as silent or clinical MI, CHF, or even SCD. Scott and collaborators described the clinical characteristics of 61 patients with healed transmural MI on necropsy of which only 33 had a clinical history of acute MI 6. In patients with MI on autopsy, DM was significantly more common in those without a clinical history of MI (46%) as compared to those with a prior history of MI (15%) 6. Similarly, the prevalence of abnormal stress imaging scans as a measure of myocardial ischemia or scar is high among asymptomatic DM individuals 7-15. Depending on the populations studied and inclusion criteria, the percentage of DM patients with abnormal scans range widely between 10% and 62% (Table 1) 13-17. De Lorenzo, et al examined 180 asymptomatic DM subjects (disease duration not specified) with rest and stress single photon emission computed tomography (SPECT) 14 for pre-operative evaluation or for screening of asymptomatic CHD. The prevalence of abnormal SPECT scans (defined as ischemia, scar or both) was 26%. Rajagopalan *et al*., performed rest and stress SPECT on 1,427 relatively high-risk, asymptomatic (mostly type 2) DM individuals (median duration 10 yrs) 14. The indications for SPECT evaluation included pre-operative assessment, screening, and non-specific symptoms (other than angina or dyspnea). Importantly, 9% of the subjects had evidence of Q-waves on electrocardiogram suggestive of prior MI. The frequency of abnormal SPECT studies was high (58%) in the study population, and 18% of patients had high-risk scans. Finally, Scognamiglio *et al.,* studied 1899 asymptomatic type 2 DM patients (duration 9.3 ±5.6 yrs) with dipyridamole myocardial contrast echocardiography (MCE) followed by invasive coronary angiography for those with positive MCE 17. Of the 60% of individuals with abnormal MCE, 65% (736/1133) had ≥50% luminal diameter narrowing of one or more major epicardial coronary arteries or major branches. Importantly, the prevalence of three-vessel CHD was 7.6% among patients with abnormal MCE and ≤1 associated risk factors, but 33.3% in those with abnormal MCE and ≥2 risk factors 17.

**Table 1.**
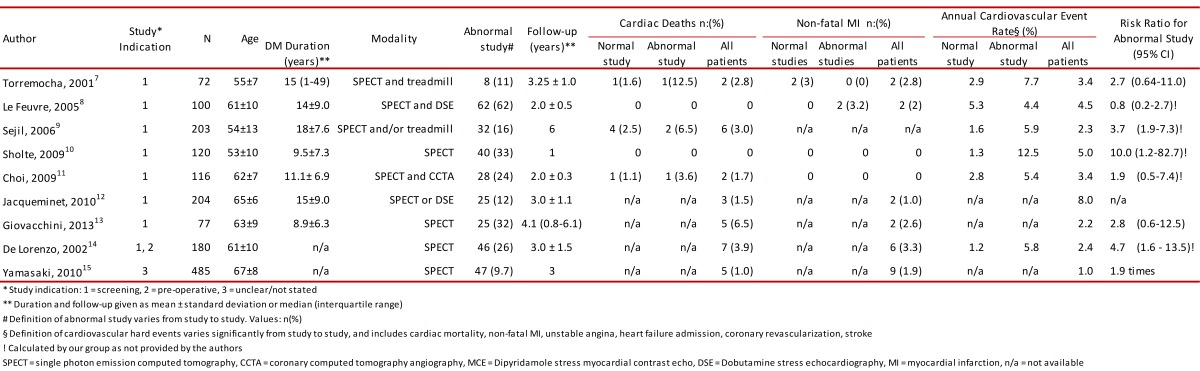
Summary of observational studies using cardiac testing for evaluation of coronary heart disease and cardiovascular outcomes in apparently asymptomatic individuals with diabetes mellitus.

The aforementioned literature highlights the relative high frequency of abnormal cardiac stress testing findings and CHD on coronary angiography among those with abnormal studies in seemingly asymptomatic individuals with DM. However, routine screening in asymptomatic individuals is not part of current guidelines and the role of testing is unclear.

## Role of screening asymptomatic diabetic individuals for coronary heart disease

The discordance between the apparently high prevalence of CHD and lack of warning symptoms, such as chest pain, has been attributed to diabetic autonomic neuropathy involving the cardiac afferent sympathetic fibers, which are a key component of cardiac pain perception. The early clinical manifestations of CHD may not be perceived or they may be atypical or non-specific. As a result, more severe presentations of ischemia --acute MI, SCD or advanced CHF-- may be the first clinical presentation of CHD in DM.

A number of observational studies have tried to investigate the potential role of CHD screening in seemingly asymptomatic DM individuals by evaluating the cardiovascular outcomes after cardiac testing that included rest/stress SPECT, treadmill ECG, stress echocardiography, and coronary computed tomography angiography (CCTA; Table 1) 07- 15. Using hard (e.g. cardiac mortality, and non-fatal MI), as well as more subjective (e.g. coronary revascularization) major adverse cardiovascular events as endpoints, most studies indicated that patients with abnormal or high risk cardiac testing had significantly higher annual rates of events compared to individuals with normal or low-risk cardiac testing (Table 1). For example, DeLorenzo, *et al.,* reported that non-fatal MI or cardiac death rates were 2% per year for normal SPECT versus 9% per year for those with abnormal SPECT scans over 3.0 ±1.5 yrs of follow up. Rajagoapalan, *et al*., reported the annual mortality rate of 5.9% for high-risk, 5.0% for intermediate-risk, and 3.6% for low-risk SPECT scans after 5.8 ±3.5 yrs of follow-up. These data suggest that asymptomatic ischemia and infarction are not uncommon and when present, portends a graded risk of cardiovascular events depending on the degree of ischemia.

The results of these, and other observational, non-randomized studies, suggest that the use of screening tools for identification of CHD in asymptomatic DM individuals may be appealing and has been recommended in certain circumstances 18. However, the main limitation of these observational cohort studies is that they are not really "testing" the role of screening and the benefit of any interventions that may follow. The optimal method of evaluating a screening test is a randomized trial.

Recently, two clinical trials have now addressed this question by randomizing asymptomatic DM patients to screening or not using cardiac SPECT 19, 20 or CCTA 21. The Detection of Ischemia in Asymptomatic Diabetics (DIAD) study was a multicenter prospective trial in the U.S. and Canada of asymptomatic patients with type 2 DM without known or suspected CHD 19. Between July 2000 and August 2002, 1,123 subjects were randomized to either screening rest/stress SPECT (n= 561) or continued on the standard of care (n= 562) and followed for approximately 5 yrs. The mean DM duration at randomization was 8.2 ±7.1 yrs in the screened group and 8.9 ± 6.9 yrs in the control group (Table 2). Preliminary findings revealed that 22% (113/522) of the screening SPECT images were consistent with silent ischemia although only 6% (n= 33) had moderate or large stress perfusion defects 19. The primary outcome of cardiac death or non-fatal MI over the mean 4.8 yrs follow up was relatively low with a cumulative cardiac event rate of 2.9% or 0.6% per year 20. Most importantly, the number of cardiac events did not differ between the screening SPECT and standard of care groups. Only 7 non-fatal MI and 8 cardiac deaths (2.7%) occurred in the screened group and 10 non-fatal MI and 7 cardiac deaths (3.0%) among the standard of care group (HR, 0.88; 95%CI, 0.44-1.88; *p*= 0.73). Coronary angiography (4.4% vs. 0.5%; *p* <0.001) and revascularization (1.6% vs. 0.36%; *p*= 0.03) were performed more frequently in the screened than control group during the first 120 days after randomization, likely as a result of abnormal SPECT scans. However, the overall rate of coronary angiography (14% vs. 12%; *p*= NS) and coronary revascularization (5.5% vs. 7.8%; *p*= 0.14) were comparable in the screened and unscreened group after the conclusion of the 5-year follow-up period. Importantly, during the course of the trial there was a significant and equivalent increase in primary prevention medical treatments, including aspirin, statins and angiotensin converting enzyme inhibitors, in both groups. Thus, data from the DIAD study suggest that screening asymptomatic type 2 DM patients with SPECT stress testing did not reduce the cardiac events compared to contemporary standard of care.

**Table 2.**
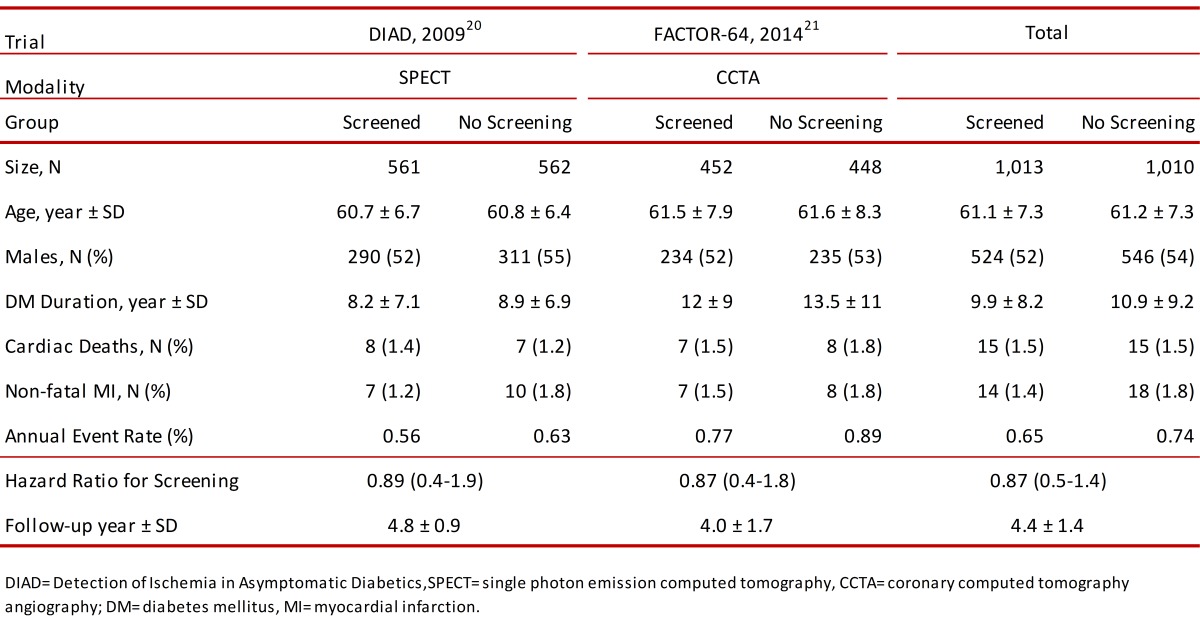
Cardiovascular outcomes after randomization of asymptomatic individuals with diabetes mellitus to screening for evaluation of coronary heart disease.

A more recent trial, the FACTOR-64 study randomized 900 high-risk, asymptomatic patients with type 1 or type 2 DM to screening CCTA scan followed by a specific treatment plan based on CCTA findings (n= 452) or to the standard of care (n= 447) 21. Mean DM duration was 12.3 ±9.23 and 13.5 ±10.7 yrs in the screened and control group, respectively. The primary composite outcome was all-cause mortality, non-fatal MI, or unstable angina. According to CCTA results, prevalence of severe (defined as ≥70% stenosis in at least 1 major proximal or large coronary artery) or moderate CHD (50% - 69% stenosis or coronary artery calcium score >100) was 6.3% (n=21), and 10.7% (n=36) respectively in the screened population. Similar to the DIAD Study, the overall proportion of patients undergoing coronary angiography (13.3% vs. 5.1%) or revascularization with percutaneous coronary intervention (6.0% vs. 1.8%) or coronary artery bypass graft surgery (2.9% vs. 1.3%) were more common in the CCTA screened population than the standard of care group. Nevertheless, at a mean follow-up time of 4.0 ±1.7 yrs, the primary composite end-point was similar in both groups (6.2 and 7.6%, respectively). A total of 16 all-cause deaths, 7 non-fatal MI and 9 hospitalizations for unstable angina (6.2%) were seen in the screened group, 19 all-cause deaths, 8 non-fatal MI and 9 hospitalizations for unstable angina (7.6%) occurred in the control group (HR, 0.80; 95%CI, 0.49-1.32; *p*= 0.38). The incidence of the composite secondary end point of ischemic major adverse cardiovascular events also did not differ between groups (4.4%: 20 events vs. 3.8%: 17 events; HR: 1.15; 95%CI: 0.60-2.19; *p*= 0.68). 

In summary, the FACTOR-64 trial showed that screening asymptomatic patients with DM using CCTA, a modality that primarily serves as an anatomical rather than functional estimation of ischemia, failed to reduce major cardiovascular events compared to the standard of care alone. 

Potential explanations for the lack of benefit of screening in these two trials are numerous. First, the sample size of both studies combined was 2,023 participants and in the presence of lower than expected annual event rates (<1.0% per year in both trials), it is conceivable that these studies may have been underpowered. For example, looking at the control (no screening) groups of DIAD (n= 562) and FACTOR-64 (n= 448), the combined number of cardiac deaths (n= 15) and non-fatal MI (n= 18) was only 33, representing a cumulative event rate of 3.3% after a mean follow-up of 4.4 yrs. Calculation of sample size will partly depend on the effectiveness of the intervention (e.g. coronary revascularization) to reduce event rates in the screening compared to the control group. Assuming 80% power, and an alpha of 0.05 for a 2-tailed test, the estimated sample size for each group would be 3,547, 3,108 and 831 individuals for an intervention with 20% (3.3% to 2.6%), 30% (3.3% to 2.3%) and 40% (3.3% to 1.98%) event reduction rates respectively. This means that the combination of both trials, theoretically, would have yielded adequate power (n= 2,023) to detect significant differences if the intervention(s) were capable of causing a 40% event reduction in the screened group. This clinical effect was obviously not seen in either trial. From this hypothetical exercise, one could argue that a significantly larger study (>6,000 subjects) would be required to detect a clinically important relative risk reduction of 20% to 30% in cardiac mortality or non-fatal MI among screened individuals. Furthermore, perhaps a longer follow-up than the mean 4.4 yrs duration for both studies could also aid detect more clinical differences between the screening and no screening groups.

Additional potential contributors to the low event rate observed may also be the types of patients enrolled in the trials. Population-based studies have reported that the duration of DM (usually of more than 10 years' duration) appears to be an important determinant in the prediction of adverse cardiovascular events, especially among DM men aged 60 and 79 22. In this respect, FACTOR-64 enrolled men ≥50 yrs or women ≥55 years with DM documented for at least 3 years or men ≥40 years or women ≥45 years with DM for at least 5 yrs, whereas, DIAD recruited patients between the ages of 50 and 75 yrs with type 2 DM onset at age ≥30 yrs. Mean DM duration was 12.7 ±10 yrs, and 8.5 ±7.0 yrs in FACTOR-64 and DIAD, respectively. Therefore, patients in the DIAD study and perhaps less so in the FACTOR-64 did not have an adequately long duration of DM to be considered "high risk" and therefore potentially benefit from revascularization. Future studies may be required to further investigate if CHD screening is suitable in a specific subset of higher-risk DM individuals without symptoms.

Another point worth discussing is the low prevalence of significant CHD in these trials. For example, in DIAD, even though 22% of the screened individuals had abnormal SPECT, only 6% of them were considered to have high-risk scans. Similarly, in FACTOR-64 only 6.3% of screened patients had CCTA evidence of severe CHD. This is an important fact as most observational studies in the literature (Table 1) suggest higher prevalence of abnormal cardiac scans and/or CHD, which is likely explained by differences in the grading systems of abnormal cardiac testing and/or definitions of CHD, as well as a wide variation in inclusion/exclusion criteria between studies. In this last aspect, clinical trials are undoubtedly more selectively performed (e.g. DIAD excluded patients with abnormal electrocardiograms), potentially minimizing the chances of enrolling people with more overt CHD.

Lastly, in the DIAD and FACTOR-64 trials, significant improvements in medical management were also seen over time. In the DIAD study, nearly 2/3 of patients, irrespective of randomization group, were taking aspirin (~73%), statins (~67%) or anti-hypertensive drugs (~74%) by the end of the 5-year follow-up period. In a similar way, in FACTOR-64, LDL (74% of patients were on statins), and systolic blood pressure were usually at goal, implying adequate medical regimens in both groups, which may have contributed significantly to the low cardiac event rates observed in both trials. These findings may be supported by the results from the COURAGE (34% had DM) and BARI 2D (all patients had type 2 DM) trials where major cardiovascular events were similar between optimal medical therapy alone or with coronary revascularization in symptomatic patients with, otherwise, stable CHD 23, 24. Although this is only hypothesis generating, the cumulative data does not suggest that coronary revascularization should be performed for all DM patients with CHD and ischemia. 

Both the DIAD and FACTOR-64 studies clearly highlight the importance of sponsoring and conducting well-designed randomized clinical trials to confirm or dispute (such as in this case) previously made assumptions from observational data that is subject to a number of inherent bias and limitations. The prospective, randomized ISCHEMIA trial of revascularization versus optimal medical therapy alone in symptomatic patients with moderate to severe ischemia may shed further light on the role of revascularization in truly higher risk patients. However, additional trials in the highest risk, asymptomatic DM patients are needed for definitive assessment of benefit or lack of benefit of screening.

## Conclusion

Screening using non-invasive cardiac imaging with either SPECT or CCTA for occult or undiagnosed CHD in asymptomatic DM individuals is not supported by two moderately sized, well-conducted, randomized trials. Given the lower than expected rates of major cardiovascular events in the trials, optimal medical therapy appears important. Additional data on the benefits in higher risk, asymptomatic DM patients with longer DM duration or with higher risk scans are needed. 
